# The Lysine Demethylase dKDM2 Is Non-essential for Viability, but Regulates Circadian Rhythms in *Drosophila*

**DOI:** 10.3389/fgene.2018.00354

**Published:** 2018-09-04

**Authors:** Yani Zheng, Yongbo Xue, Xingjie Ren, Mengmeng Liu, Xiao Li, Yu Jia, Ye Niu, Jian-Quan Ni, Yong Zhang, Jun-Yuan Ji

**Affiliations:** ^1^Department of Molecular and Cellular Medicine, College of Medicine, Texas A&M University Health Science Center, College Station, TX, United States; ^2^Department of Biology, University of Nevada, Reno, Reno, NV, United States; ^3^Gene Regulatory Laboratory, School of Medicine, Tsinghua University, Beijing, China

**Keywords:** KDM2, demethylase, development, circadian rhythms, *Drosophila*

## Abstract

Post-translational modification of histones, such as histone methylation controlled by specific methyltransferases and demethylases, play critical roles in modulating chromatin dynamics and transcription in eukaryotes. Misregulation of histone methylation can lead to aberrant gene expression, thereby contributing to abnormal development and diseases such as cancer. As such, the mammalian lysine-specific demethylase 2 (KDM2) homologs, KDM2A and KDM2B, are either oncogenic or tumor suppressive depending on specific pathological contexts. However, the role of KDM2 proteins during development remains poorly understood. Unlike vertebrates, *Drosophila* has only one KDM2 homolog (dKDM2), but its functions *in vivo* remain elusive due to the complexities of the existing mutant alleles. To address this problem, we have generated two *dKdm2* null alleles using the CRISPR/Cas9 technique. These *dKdm2* homozygous mutants are fully viable and fertile, with no developmental defects observed under laboratory conditions. However, the *dKdm2* null mutant adults display defects in circadian rhythms. Most of the *dKdm2* mutants become arrhythmic under constant darkness, while the circadian period of the rhythmic mutant flies is approximately 1 h shorter than the control. Interestingly, lengthened circadian periods are observed when dKDM2 is overexpressed in circadian pacemaker neurons. Taken together, these results demonstrate that *dKdm2* is not essential for viability; instead, dKDM2 protein plays important roles in regulating circadian rhythms in *Drosophila*. Further analyses of the molecular mechanisms of dKDM2 and its orthologs in vertebrates regarding the regulation of circadian rhythms will advance our understanding of the epigenetic regulations of circadian clocks.

## Introduction

Covalent histone modifications, particularly methylation and acetylation of lysine (K) residues on the N-terminal tails of histones H3 and H4, play fundamental roles in epigenetic regulation of gene expression in eukaryotes. The addition or removal of methyl groups to lysine residues is dynamically modulated by specific lysine methyltransferases (KMTs) and lysine demethylases (KDMs), respectively (Shi and Whetstine, 2007; Black et al., 2012). These enzymes are highly conserved in the evolution of eukaryotes (Zhou and Ma, 2008; Zhang and Ma, 2012; Qian et al., 2015). Extensive efforts have been devoted to elucidating the molecular mechanisms of how these enzymes catalyze reactions toward specific lysine residues (Shi and Whetstine, 2007; Black et al., 2012). Based on the chemical reactions that they catalyze, the KDMs are classified into two major families: the lysine-specific histone demethylase (LSD) family, including LSD1 (also known as KDM1) and LSD2, and the Jumonji C (JmjC) domain family demethylases, containing more than 20 KDMs in mammals (Shi and Whetstine, 2007; Shi and Tsukada, 2013). The LSD family demethylases can only demethylate mono- and dimethylated (me1 and me2) lysine residues through an FAD-dependent amine oxidase reaction; the JmjC domain family demethylases can demethylate me1, me2, and trimethylated (me3) lysine residues through a 2-oxoglutarate-Fe(II)-dependent dioxygenase reaction (Shi and Whetstine, 2007; Cloos et al., 2008; Shi and Tsukada, 2013). Since the initial discovery of LSD1 (Shi et al., 2004), we have witnessed remarkable progress in the identification and characterization of these demethylases in diverse organisms, particularly in their substrate specificities and their effects in regulating transcription at single-gene and genome-wide levels. However, the *in vivo* function and regulation of these enzymes in developmental, physiological, and pathological contexts are still not fully understood (Nottke et al., 2009; Dimitrova et al., 2015).

Based on their sequence homology and their specificities in removing the methylation marker on lysine residues, the JmjC KDMs (KDM2-7) are further grouped into five clusters, including KDM2/7, KDM3, KDM4, KDM5, and KDM6 (Klose et al., 2006; Cloos et al., 2008). In vertebrates, the KDM2 subfamily of KDMs consists of KDM2A and KDM2B; studies in the past decade have linked mutations of these KDM2 paralogs to a number of human cancers (Klose et al., 2006). For example, KDM2A has been reported to play an oncogenic role in breast cancer (Liu et al., 2016), and overexpression of KDM2B and its oncogenic role has also been observed in acute myeloid leukemia (He et al., 2011) and pancreatic cancer (Tzatsos et al., 2013). However, KDM2A appears to play a tumor suppressive role in colon cancer (Lu et al., 2010), and similarly, KDM2B is implicated to play a tumor suppressor role in lymphoma and brain cancer (Suzuki et al., 2006; Frescas et al., 2007). Thus, the role of KDM2A and KDM2B in tumorigenesis seems to be dependent on different types of cancers. Elucidation of the *in vivo* functions of these enzymes is key to understanding how misregulation of KDM2 proteins contributes to these different cancers.

To address this problem, we have performed developmental genetic analyses of *Kdm2* mutants in *Drosophila* because of its relative simplicity compared to mammals. *Drosophila* also provides sophisticated genetic tools to dissect the function and regulation of genes of interest. *Drosophila* has 13 KDMs, with dKDM2 being the only one conserved KDM2 ortholog (Lagarou et al., 2008; Kavi and Birchler, 2009; Zheng et al., 2014; Holowatyj et al., 2015). Based on analyses of 13 mutant alleles of *dKdm2* gene, we have concluded that *dKdm2* is not required for normal development in *Drosophila* (Zheng et al., 2014). This notion is unexpected for three reasons. First, dKDM2 has been identified as a subunit of the dRING-associated factor (dRAF) complex, a Polycomb group (PcG) complex that was proposed to play critical roles in coordinating trans-histone regulation by replacing the active H3K36me2 marker with a repressive mono-ubiquitinated H2A K118 (H2AK118ub or H2Aub) marker (Lagarou et al., 2008). Second, dKDM2 was shown to demethylate H3K36me2 but not H3K36me1/3 or H3K4me3 in *Drosophila* S2 cells (Lagarou et al., 2008), but depletion of dKDM2 in *Drosophila* larvae specifically increased the levels of H3K4me3 but not H3K36me2 (Kavi and Birchler, 2009). Third, the dRAF complex has been reported to be required for producing H2AK118ub in *Drosophila* (Lagarou et al., 2008), yet a recent report demonstrates that the bulk of H2AK118ub is produced by the RING1-L(3)73Ah complex instead (Kahn et al., 2016).

A major concern in our previous genetic analyses is that all existing mutant alleles of the *dKdm2* locus have their limitations: the transposon insertion lines are either hypomorphic or fail to affect the mRNA and protein levels of dKDM2, and the three deficiency lines that uncover the *dKdm2* locus disrupt both *dKdm2* and its neighboring genes (Zheng et al., 2014). Thus having a molecularly defined *dKdm2* null allele that only disrupts the *dKdm2* locus is required to resolve the aforedescribed contradictory observations, and will enable us to draw stronger conclusions about the *dKdm2* null phenotypes and its molecular functions.

Using the CRISPR-Cas9 technique, we have generated two null alleles that only disrupt *dKdm2* but not its neighboring genes. Our analyses of these two null alleles demonstrate that the *dKdm2* gene is indeed not required for normal development, viability, or fertility. The methylation states on H3K36 and H3K4 are mildly elevated in the homozygous mutant larvae, but no effect on H2Aub was observed in these mutants. In contrast, overexpression of wild-type dKDM2 in larvae or wing discs only reduced H3K36me2 and to a lesser extent H3K36me1, but not the levels of H3K36me3. Although the *dKdm2* homozygous mutants are fully viable, the mutant flies have defective circadian rhythms; their circadian clocks become arrhythmic and have a shorter circadian period than the control. Consistent with this observation, overexpression of *dKdm2* in circadian neurons lengthens the circadian period. Taken together, our results show that dKDM2 regulates circadian rhythms, but does not play any roles essential for survival in laboratory settings during normal development.

## Materials and Methods

### Fly Strains

*Drosophila* strains were maintained on standard cornmeal-molasses-yeast food at 25°C, and the *w^1118^* line served as the control. The following Gal4 lines were obtained from the Bloomington *Drosophila* Stock Center: *engrailed-Gal4* (*en-Gal4*; BL-1973), *Sgs3-Gal4* (BL-6870), and *ubiquitin-Gal4* (*Ubi-Gal4*; BL-32551).

### Generation of the *UAS-dKdm2^+^-eGFP* Transgenic Line

The *dKdm2* cDNA was subcloned to the pENTR vector using the pENTR/D-TOPO Cloning kit (Invitrogen, K240020). The pTWG vector (from the DGRC #1076^1^) was used as the destination vector and the *att*L x *att*R reaction was mediated by the LR Clonase II enzyme mix (Invitrogen, 11791-020), resulting in the ‘*pUASt-dKdm2^+^-eGFP*’ transgenic vector. After injecting the vector into *w^1118^* embryos, the *UAS-dKdm2^+^-eGFP* transgenic lines were established by standard fly genetics.

### Generation and Validation of the *dKdm2* Null Alleles *dKdm2^1^*and *dKdm2^2^*

Two *dKdm2* null alleles were generated using the *Drosophila* germline specific CRISPR/Cas9 system (Ren et al., 2013, 2014). Four sgRNAs were designed to target coding regions of *dKdm2* (NO3: 5′-AGCTGGTAGAGCGAAGGCGG-3′, NO4: 5′-GAGGATGGCGAGGGGACGCG-3′, NO5: 5′-TCTTCAAAGTTCGCGCAGGC-3′, NO6: 5′-TACATTGCTGCCGCCCCCGG-3′) and cloned into the U6b-sgRNA plasmid. To generate *dKdm2* null alleles, sgRNA plasmids (sgRNA3, 4, 5 for *dKdm2^1^*, sgRNA4, 5, 6 for *dKdm2^2^*) were injected into *nos-Cas9 Drosophila* embryos. All adult flies developed from injected embryos were crossed with the “*y^1^ sc^1^ v^1^; +; Dr^1^ e^1^/TM3 Sb^1^*” strain, and null alleles were screened in the F1 generation with single crossing and *Drosophila* wing genomic DNA PCR. *dKdm2^1^* and *dKdm2^2^* stable homozygous lines were established with standard crossing protocol.

To further validate these mutant alleles, we extracted genomic DNA from 10 homozygous *dKdm2^1^*and *dKdm2^2^* mutant larvae using DNAzol (Invitrogen, 10503-027). PCR was performed with Taq polymerase (Invitrogen) for 35 cycles (elongation for 5.5 min and annealing at 55°C), as described previously (Zheng et al., 2014). The following primers were used: *dKdm2-3F*: 5′-CGGTTGTAGCCGTTAGGAAA-3′; and *CRISPR dKdm2-3.1*: 5′-TTTGCATTGCGTGTGGTTAT-3′. The PCR products were further validated by sequencing.

### Generation of the *dKdm2^+^-eGFP* Line

To tag the endogenous *dKdm2* locus with eGFP, we used the germ-line specific CRISPR/Cas9 system (Ren et al., 2013, 2014). One sgRNA was designed to target 3′ UTR of *dKdm2* (CTGGCAGCTGGAGGAGGGCC) and cloned into the U6b-sgRNA plasmid. The *dKdm2^+^-eGFP* donor construct was based on the pBluescript plasmid. The left homologous arm of *dKdm2* was amplified from genomic extract with *dKdm2*-*left-F* primer (5′-TCTCTCAGTTGGGGAAGCTTCCACTAATCAGTGGAGTGGCAGTGGC-3′) and *dKdm2-left-R* primer (5′-TCCGCTTCCTCCTCCTCCGCTTCCACCTCCTCCGTCGTGGTGCCAGCTTCGGT-3′). The right homologous arm was amplified with *dKdm2-right-F* primer (5′-GCGACTGGCAGCTGGAGGAGGGC-3′) and *dKdm2-right-R* primer (5′-TCTCTCAGTTGGGGAAGCTTAAGATGCAGGACGGTCGCAAGACG-3′). The gene encoding the *eGFP* was amplified with *eGFP-F* primer (5′-GGAGGAGGAGGAAGCGGAGGAGGAGGTAGCGTGAGCAAGGGCGAGGAGCTG-3′) and *eGFP-R* primer (5′-CTCCTCCAGCTGCCAGTCGCTTACTTGTACAGCTCGTCCATGCCGAG-3′). A GS linker was synthesized in *eGFP-F* and *dKdm2-left-R* primers. These fragments were stitched together using sequence- and ligation-independent cloning (Jeong et al., 2012). All PCR fragments were amplified with KOD DNA polymerase (TOYOBO). The donor construct was confirmed by sequencing with *eGFP-seq-R* primer (5′-ACCCCGGTGAACAGCTCCT-3′) and *dKDM2-seq-F* primer (5′-GCGGCGACTCCAAAGTAGGAAA-3′). To generate *dKdm2-eEFP* flies, sgRNA plasmids and donors were co-injected into nos-Cas9 *Drosophila* embryos. All G0 adult flies that developed from injected embryos were crossed with the “*y^1^ sc^1^ v^1^; +; Dr^1^ e^1^/TM3 Sb^1^*” line, and knock-in events were screened by PCR of F1 adults and validated by sequencing. The *dKdm2-eGFP* stable homozygous lines were established using standard fly genetics.

### Characterization of the *dKdm2* Alleles Using Western Blot and qRT-PCR

The Western blot analyses of the *dKdm2* alleles were performed as described (Zheng et al., 2014). A Li-Cor Odyssey Infrared Imaging system was used to quantify the Western blots shown in **Figures 2**, **3**. The primary antibodies used were described previously (Zheng et al., 2014), and the following secondary antibodies from Li-Cor Biosciences are used in these analyses: IRDye 800CW Goat anti-Rabbit IgG (926-32211) and IRDye 680 Goat anti-Mouse IgG (926-32220). At least three independent biological repeats were quantified in each experiment. The primers and conditions for qRT-PCR assays were the same as described previously (Zheng et al., 2014). Statistical significance was determined using one-tailed Student’s *t*-test.

### Immunostaining Procedure of Wing Discs

Wing discs from the third-instar wandering larvae were dissected in phosphate buffered saline (PBS), and then fixed in 5% formaldehyde in PBS for 10 min at room temperature. After rinsing with PBT (PBS with 0.2% Triton X-100), the discs were blocked with PBTB (PBT with 0.2% BSA and 5% normal goat serum) for 1 h at room temperature with rocking, and then incubated with the following primary antibodies. Anti-H3K4me2 was purchased from Cell Signaling Technology (#9725; 1:500 diluted in PBTB). The following antibodies were purchased from Abcam: anti-H3K4me1 (ab8895; 1:2500), anti-H3K4me3 (ab8580; 1:500), anti-H3K36me1 (ab9048; 1:500), anti-H3K36me2 (ab9049; 1:1000), and anti-H3K36me3 (ab9050; 1:500). After rinsing, the discs were then incubated with the secondary antibody (1:500 in PBTB) for 1 h at room temperature on a nutator. Finally, the discs were rinsed with PBT and mounted with VECTASHIELD. Confocal images were collected using a Nikon Ti Eclipse microscope, and then processed using the Adobe Photoshop CS6 software.

### Analyses of the Circadian Locomotor Behaviors

Two to five days old male flies were collected and loaded into *Drosophila* Activity Monitor (DAM, TriKinetics). Flies were entrained to 12 h light: 12 h dark cycles (LD) at 25°C for 4–5 days and then released into constant darkness (DD) for 7 days. Locomotor activity rhythms were analyzed using FaasX software. Average actograms were generated by MATLAB with Griffith sleep analysis toolbox, which was kindly shared from Fang Guo at Michael Rosbash’s lab.

### Whole-Mount Brain Immunohistochemistry

Adult flies were entrained in LD for 4–5 days, and then they were collected at the indicated circadian time (CT) on the first day of DD. Flies were immediately fixed in 4% paraformaldehyde in PBS at room temperature for 1 h and further dissected in PBS containing 0.1% Triton-X100 (PBST). After three washes using PBST, fly brains were blocked in PBST with 10% normal goat serum at room temperature for 1 h. Next, they were incubated at 4°C overnight with primary antibodies: rabbit anti-PER (1:5000), guinea pig anti-CLK (1:2500), and mouse anti-PDF (C7, Developmental Studies Hybridoma Bank, 1:400). Secondary antibodies were diluted at 1:200 concentration with respect to anti-mouse 488, anti-rabbit 594, and anti-guinea pig Cy3 (Jackson Immuno Research). For dKDM2-EGFP expression, brains were dissected and stained with an anti-GFP antibody (Invitrogen, A6455; 1:200) and mouse anti-PDF (1:400). They were then imaged using a Leica TCS SP8 confocal microscope and the images were quantified using NIH Image^2^. Statistical significance was determined by two-tailed Student’s *t*-test at *p* < 0.05.

### Data Availability Statement

All strains and plasmids are available upon request. The authors affirm that all data necessary for confirming the conclusions of the article are present within the article, figures, and tables.

## Results

### Generation of the *dKdm2* Null Alleles Using the CRISPR/Cas9 Technique

**FIGURE 1:**
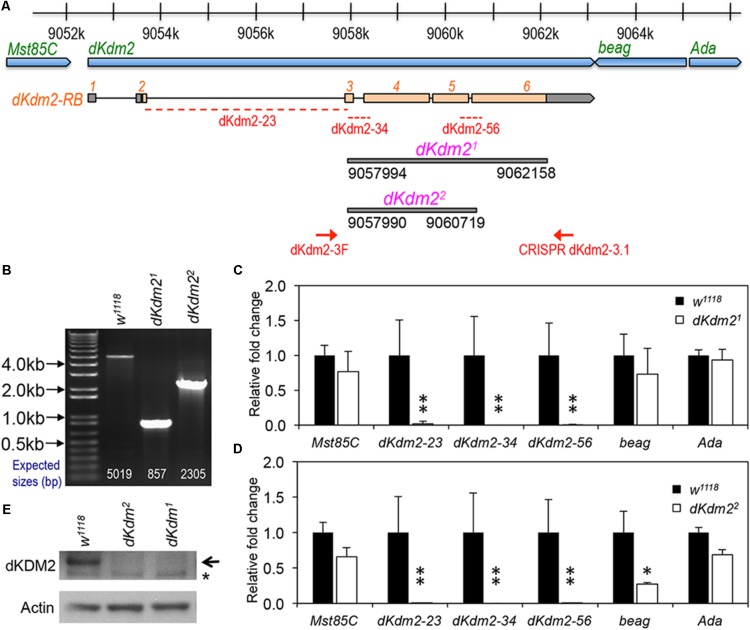
Generation and characterization of the *dKdm2* null alleles. **(A)** Schematic drawing of the *dKdm2* locus and its neighboring genes, note the regions that are deleted in *dKdm2^1^* and *dKdm2^2^* and the corresponding breakpoints. **(B)** Validation of the *dKdm2* alleles using PCR and genomic DNA. The positions of the primers (“dKdm2-3F” and “CRISPR dKdm2-3.1”) are shown in **(A)**. **(C,D)** The mRNA levels of *dKdm2* and the neighboring genes *dKdm2*, *Mst85C*, *beag*, and *Ada* in the third instar larvae are measured by qRT-PCR assay. The positions of the primers for three regions within the *dKdm2* locus are shown in **(A)**. ^∗^*P* < 0.05, ^∗∗^*P* < 0.01 based on Student’s *t*-tests (one-tailed). **(E)** The protein levels of dKDM2 in the third instar (L3) wandering larvae were analyzed by Western blot. The non-specific bands are marked with ‘^∗^,’ and anti-actin was used as the control.

To determine the role of dKDM2 in *Drosophila* development, we generated two mutant alleles that only delete the coding region of *dKdm2*, designated as *dKdm2^1^* and *dKdm2^2^* (**Figure 1A**), using the CRISPR/Cas9 technique (Ren et al., 2014; Bier et al., 2018). These two alleles are null alleles of the *dKdm2* gene based on the following evidence. First, by performing PCR using the genomic DNA from the third instar larvae of these two mutant lines, we observed shorter PCR products in the mutants compared to the control (**Figure 1B**). Second, sequencing the PCR products revealed that *dKdm2^1^* deletes the region 9057994∼9062158 of the third chromosome, and *dKdm2^2^* deletes the region 9057990∼9060719 (**Figure 1A**). Third, the transcripts of *dKdm2* are undetectable as analyzed by qRT-PCR (**Figures 1C,D**). Furthermore, the dKDM2 protein is not detectable from the mutant larvae as assayed by a Western blot (**Figure 1E**). These analyses demonstrate that *dKdm2^1^* and *dKdm2^2^* disrupt the *dKdm2* locus. For unknown reasons, the mRNA level of *beag*, a neighboring gene of *dKdm2*, is also reduced in the *dKdm2^2^* mutants, as assayed by multiple repeats. Nevertheless, our analyses with both these alleles did not reveal any difference between them (see below). These results show that both *dKdm2^1^* and *dKdm2^2^* are null alleles.

Importantly, the homozygous mutants of *dKdm2^1^* and *dKdm2^2^*, as well as the transheterozygous animals (genotype: *w^1118^; +; dKdm2^1^/dKdm2^2^*), are fully viable and fertile, and do not have any developmental defects. In fact, the homozygous mutants of *dKdm2^1^* and *dKdm2^2^* can be maintained as stable and healthy stocks for many generations. Taken together, these results demonstrate that *dKdm2* is not required for normal development. This conclusion is consistent with our previous work (Zheng et al., 2014), as well as a recent report about a null *dKdm2* allele independently generated by an ends-out gene targeting approach (Shalaby et al., 2017).

### Effects of Loss of *dKdm2* on Histone Methylation and Ubiquitination

**FIGURE 2:**
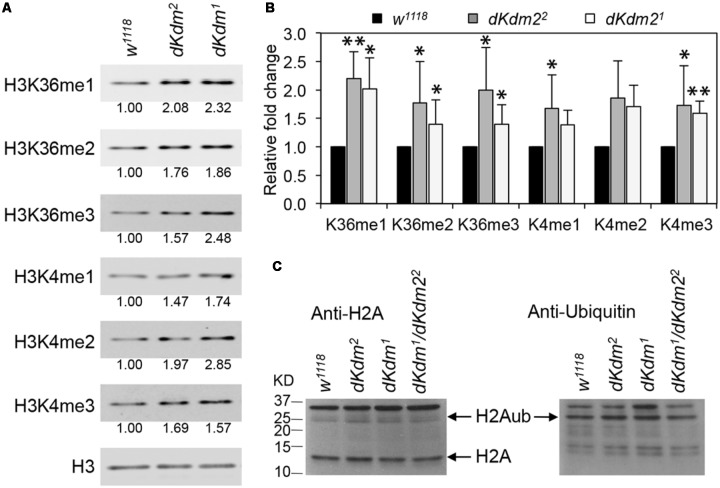
Levels of histone methylation and ubiquitination in the *dKdm2* mutants. **(A)** The levels of H3K36me1/2/3 and H3K4me1/2/3 in the *dKdm2* mutants at the L3 wandering stage. The results of Western blot **(A)** from three independent biological repeats were quantified using a Li-Cor Odyssey Infrared Imaging system **(B)**. ^∗^*p* < 0.05; ^∗∗^*p* < 0.01 based on Student’s *t*-tests. **(C)** The levels of histone H2A and ubiquitinated H2A (H2Aub) in *dKdm2* mutants at the L3 wandering stage.

Previously, we reported that methylation on H3K36 and H3K4 are only weakly affected in the deficiency and transposon insertion lines in the *dKdm2* locus (Zheng et al., 2014). With these two new *dKdm2* null alleles, we re-examined whether the methylation status of H3K36 and H3K4 were affected in the *dKdm2* null mutants using quantitative Western blot analyses. As shown in **Figure 2A**, the levels of H3K36me1/2/3 and H3K4me1/2/3 are elevated in the *dKdm2^1^* or *dKdm2^2^* homozygous mutants at the L3 wandering stage. Quantification of the Western blots from three independent experiments shows that the increase of H3K36me1/2/3 and H3K4me1/3 are statistically significant (**Figure 2B**). These results are consistent with the previous reports that dKDM2 may be involved in regulating the demethylation of H3K4 and H3K36 (Lagarou et al., 2008; Kavi and Birchler, 2009; Zheng et al., 2014).

Depletion of dKDM2 in S2 cells was shown to significantly increase H2AK118ub (Lagarou et al., 2008), and it has been reported that the mouse KDM2B is required for H2AK119ub (Wu et al., 2013). However, a recent report has identified the RING1-L(3)73Ah complex, which does not contain dKDM2, as the major regulator of H2AK118ub in *Drosophila* (Kahn et al., 2016). Thus we asked whether the levels of H2A ubiquitination was altered in the *dKdm2* null mutant larvae. As shown in **Figure 2C**, the ubiquitin levels on H2A are not affected in the *dKdm2^1^*, *dKdm2^2^*, or *dKdm2^1^*/*dKdm2^2^* transheterozygous mutant larvae at the wandering stage. This observation does not support the role of dKDM2 in regulating H2A ubiquitination. Taken together, these results suggest that the major role of dKDM2 is to regulate the methylation states of H3K36 and H3K4.

**FIGURE 3:**
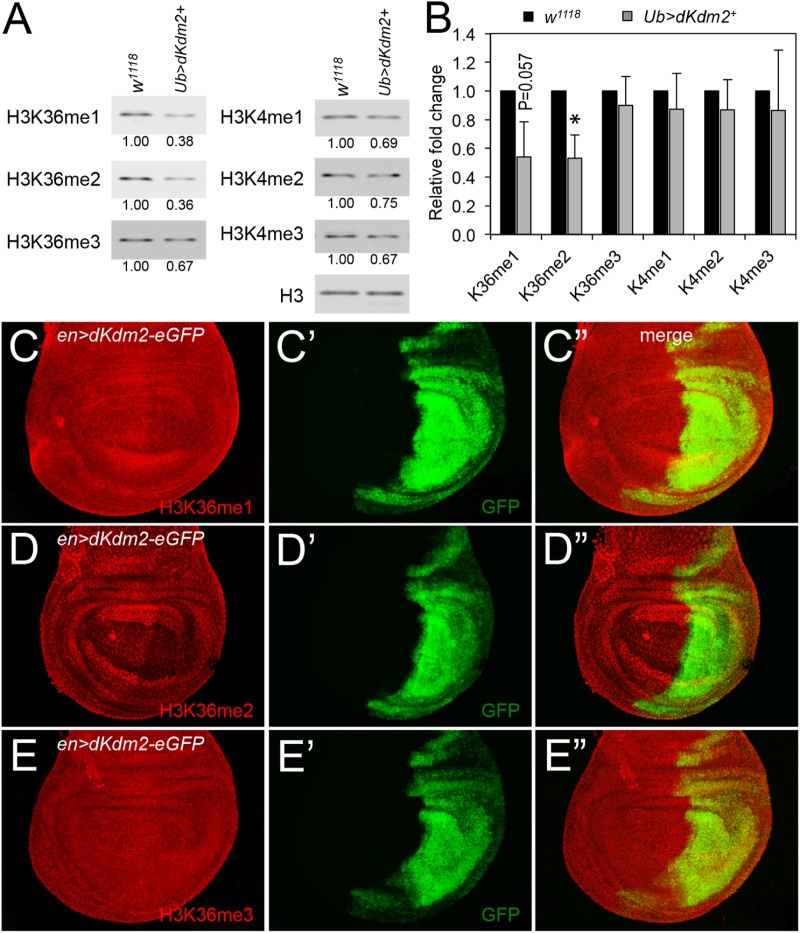
Effects of dKDM2 over-expression on methylation levels of H3K4 and H3K36. **(A)** The levels of H3K36me1/2/3 and H3K4me1/2/3 in dKdm2-overexpressed larvae at the L3 wandering stage. The detailed genotype for “*Ub > dKdm2^+^*” line is “*w^1118^; Ubi-Gal4/UAS-dKdm2-eGFP; +.*” **(B)** Quantification of the results of Western blots from three independent biological repeats using a Li-Cor Odyssey Infrared Imaging system. ^∗^*P* < 0.05 based on Student’s *t*-test. **(C–E)** Immunostaining of wing discs with wild-type *dKdm2* overexpressed in the posterior half of the discs (genotype∖*n-Gal4/UAS-dKdm2-eGFP*) with antibodies against H3K36me1 **(C)**, H3K36me2 **(D)**, and H3K36me3 **(E)**. Images in **(C′–E′)** show the GFP signals, and **(C″–E″)** are merged images.

To further analyze the role of dKDM2 on histone modifications, we asked whether over-expression of wild-type dKDM2 could have opposite effects on H3K36 and H3K4 methylation status. To test this, we generated *UAS-dKdm2* transgenic lines, which allowed us to overexpress wild-type *dKdm2* in a tissue-specific or developmental stage-specific manner (**Supplementary Figure S1**). We ubiquitously expressed dKDM2 using the *ubi-Gal4* driver and then analyzed histone methylation in L3 wandering larvae with Western blots. As shown in **Figure 3A**, overexpression of dKDM2 reduced levels of H3K36me1 (slightly) and H3K36me2 (significantly), but no obvious effects were observed regarding levels of H3K36me3 and H3K4me1/2/3. This notion is confirmed by the quantitative Western blot analyses based on three independent repeats (**Figure 3B**). These observations suggest that increasing the level of dKDM2 is sufficient to reduce H3K36me1 and H3K36me2 levels.

To complement these biochemical analyses, we performed immunostaining using *en-Gal4* to ectopically express wild-type dKDM2. The *en-Gal4* driver is expressed in the posterior compartment of the wing discs (**Figures 3C′–E′**), allowing us to compare cells in the posterior compartment with cells in the anterior compartment of the same disk. As shown in **Figure 3C**, the level of H3K36me1 is barely reduced by the overexpression of wild-type dKDM2 in the posterior compartment of the wing disc (**Figure 3C′**). In contrast, the level of H3K36me2 is clearly reduced in cells with overexpression of wild-type dKDM2 (**Figures 3D,D′**), but no regional difference was observed with H3K36me3 (**Figure 3E**) or H3K4me1/2/3 (**Supplementary Figure S2**). These observations are consistent with the biochemical analyses (**Figures 3A,B**). Taken together, these results suggest that H3K36me2 is the major target of dKDM2 during the larval stage.

### *dKdm2* Mutants Are Defective in Circadian Rhythms

Considering the high evolutionary conservation of the KDM2 family of enzymes and the fundamental roles of H3K36 methylation in transcription, it is puzzling that *dKdm2* null mutants do not display any developmental defects (Zheng et al., 2014). One clue may be obtained by analyzing the expression of the endogenous dKDM2 proteins, but this effort is hampered by the lack of the specific antibodies suitable for the immunostaining of dKDM2. After our initial report on the 10 transposon insertion lines within the *dKdm2* locus, we learned that another insertion line *dKdm2^36Y^* (*w^∗^; p[GawB]dKdm2^36Y^*) in the *dKdm2/CG11033* locus became available. This Gal4 enhancer trap line, also known as *36Y-Gal4*, is caused by the insertion of the *p[GawB]* transposon in the first intron of the *dKdm2* locus (Taghert et al., 2001). At the adult stage, this Gal4 line is expressed in the central brain, optic lobes, ring gland, salivary glands, fat body, epidermis, and hindgut (Taghert et al., 2001). *36Y-Gal4* is expressed in peptidergic neurons of the CNS and interestingly, *36Y-Gal4* driven expression of the amidating enzyme PHM affects the circadian rhythms in *Drosophila* (Taghert et al., 2001). In addition, KDM2A/FBXL11 has been identified from a screen for F-box proteins that are required for normal circadian rhythms assayed by the expression of a clock reporter in cultured human osteosarcoma U2OS cells (Reischl and Kramer, 2015). Therefore, we asked whether dKDM2 is involved in regulating circadian locomotor rhythms.

There are four large ventral lateral neurons (lLNvs) and four small ventral lateral neurons (sLNvs) that express the neuropeptide pigment dispersing factor (PDF) in each fly brain hemisphere (Nitabach and Taghert, 2008). The PDF positive sLNvs are the master pacemaker neurons that control the circadian rhythm in constant darkness (DD) (Renn et al., 1999). Although *36Y-Gal4* is expressed in the lLNvs and sLNvs, it remains unknown if the expression pattern of *36Y-Gal4* precisely represents the expression pattern of the *dKdm2* gene (Taghert et al., 2001). Thus to test whether the endogenous dKDM2 protein is expressed in adult brains, we inserted an eGFP tag in the C-terminus of the endogenous *dKdm2* locus using the CRISPR-Cas9 technique (see section “Materials and Methods”). The *dKdm2-eGFP* flies are fully viable, and we have observed that dKDM2 is expressed in adult brains (**Supplementary Figure S2A**), including the sLNvs (**Supplementary Figure S2B**) and lLNvs (**Supplementary Figure S2C**). The dKDM2 protein is predominantly localized in the nucleus (**Supplementary Figure S2**), consistent with its function of regulating histone demethylation and our previous report (Zheng et al., 2014).

**FIGURE 4:**
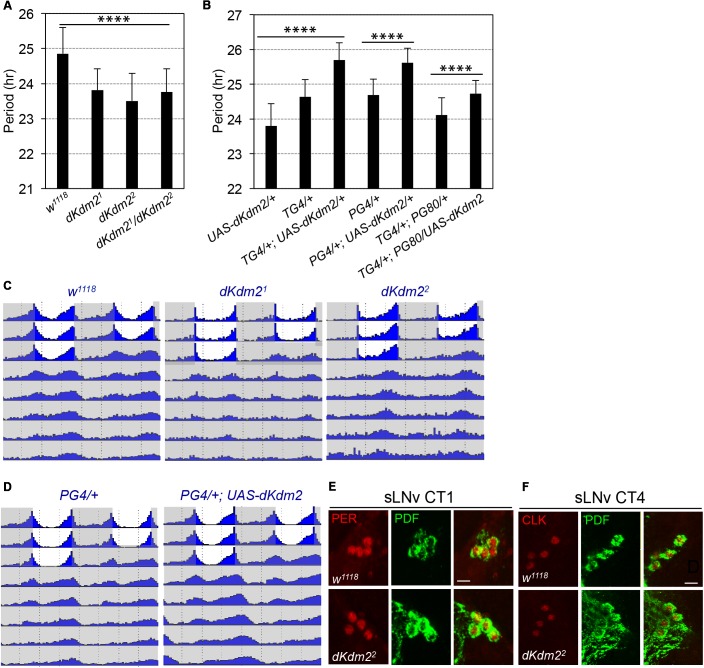
dKDM2 is required for circadian behavior rhythms. **(A)** Period of circadian locomotor rhythms of the *dKdm2* mutants. **(B)** Effects of *dKdm2* overexpression on the period of circadian locomotor rhythms. Error bars indicate SD; the detailed genotype of “*TG4/+*” is “*tim-GAL4, UAS-dicer2/+*”; “*PG4/+*” is “*Pdf-GAL4, UAS-dicer2/+*”; and “*PG80*” is “*Pdf-GAL80.*” Each genotype is compared to its driver or UAS line as the controls. For the data shown in **(A)** and first three columns in **(B)**, ^∗∗∗∗^*p* < 0.0001, as determined by using Tukey’s multiple comparison test after one-way analysis of variance. For the rest of the data shown in **(B)**, ^∗∗∗∗^*p* < 0.0001, based on *t*-test. Number of animals analyzed is included in **Supplementary Table S1**. **(C,D)** Locomotor behavior under light dark (LD) cycle and constant darkness (DD). Representative double plotted actograms showing average activity of flies during 3 days of LD and 5 days of DD for controls, *dKdm2* mutants **(C)** and *dKdm2* overexpression **(D)** in PDF positive sLNvs using the *Pdf-GAL4* driver. The light phase is shown in white color, and the dark phase is shown in gray. Representative confocal images showing the effects of *dKdm2* mutation on the levels of PER **(E)** and CLK **(F)** in sLNvs. Flies were entrained under 3 days of LD, and brains were dissected at CT0 (CT = circadian time) in the first day of DD. Fly brains were dissected at CT4. Scale bars in **(E,F)**: 10 μm.

Encouraged by these observations, we analyzed the circadian behaviors of the *dKdm2^1^* or *dKdm2^2^* homozygous mutants. They displayed defects of circadian locomotor rhythms under DD. For *dKdm2^1^*, only ∼37% of the flies were rhythmic, but the circadian period was shortened by ∼1 h (**Figure 4A** and **Supplementary Table S1**). Similar phenotypes were also observed in *dKdm2^2^* mutants, as well as the transheterozygous animals (**Figures 4A,B**). When we further analyzed the locomotor behavior under the light/dark (LD) cycle, we identified that the morning anticipatory behavior peak was also blunted in *dKdm2^1^* or *dKdm2^2^* mutants (**Figure 4C**). When we overexpressed wild-type dKDM2 in all circadian neurons using the *tim-Gal4* driver, the period was increased by ∼1 h compared to the controls (**Figure 4B** and **Supplementary Figure S3** and **Table S1**). Given that *36Y-Gal4* has weak expression in the sLNvs, we further tested the circadian behavior of flies with dKDM2 overexpression in sLNvs using the *pdf-Gal4* driver, and we observed a similar lengthened circadian period phenotype (**Figures 4B,D**). However, a weak but significant period lengthening was also observed when we overexpressed wild-type dKDM2 in all circadian tissues except the sLNvs (TG4; PG80, **Figure 4B** and **Supplementary Table S1**). We could not exclude the possibility that dKDM2 was still overexpressed in the sLNvs due to the effectiveness of the GAL80 inhibition. These results suggest that dKDM2 regulates circadian periods in sLNvs, but it may also fine-tune the rhythms in PDF negative circadian neurons.

The core of the circadian clock contains transcriptional translational feedback loops, which are highly conserved among species (Hardin and Panda, 2013). In *Drosophila*, a transactivator complex formed by CLOCK (CLK) and CYCLE (CYC) generates rhythmic transcription of hundreds of genes, including some transcriptional repressors; PERIOD (PER) is the major repressor, which accumulates in the cytoplasm, undergoes a series of post-translational modifications, and enters the nucleus to block its own transcription (Hardin and Panda, 2013). To understand how dKDM2 might affect circadian behavior, we compared protein abundance of PER and CLK at the point of their peak expression under constant darkness, circadian time 1 (CT1) for PER (circadian time) and CT4 for CLK, respectively. No obvious changes of PER (**Figure 4E**, quantified in **Supplementary Figure S4A**) and CLK (**Figure 4F** and **Supplementary Figure S4B**) levels were observed in either sLNvs, or lLNvs (**Supplementary Figures S4D,D′,E,E′**). We observed a weak but significant increase of PDF level in sLNvs for *dKdm2^2^* mutants (**Supplementary Figure S4C**). Overexpression of PDF or hyperactivation of the signaling activated by the PDF receptor is known to cause period lenthening (Wulbeck et al., 2008; Choi et al., 2009; Yoshii et al., 2009; Zhang and Emery, 2013). Thus, the increase of PDF abundance cannot explain the arrythmicity and short period phenotype of the *dKdm2^2^* mutants. These observations indicate that dKDM2 does not affect the abundance of the core pacemaker proteins. Taken together, these results show that dKDM2 is involved in regulating circadian rhythms, and varying its dosage affects the circadian rhythmicity and period.

## Discussion

Characterization of the phenotypes of different mutant alleles that disrupt the functions of a specific gene is essential to understand how its product functions during the development of an organism. In this study, we report the generation and characterization of two null alleles of the *dKdm2* gene that specifically disrupt *dKdm2* locus but not its neighboring genes. The homozygous mutant animals are fully viable and fertile, but their circadian rhythms are defective. In addition, the null mutant larvae display mild effects on methylation states on H3K36 and H3K4, but has no effect on the levels of H2Aub. Moreover, overexpression of wild-type dKDM2 in larvae and wing discs reduced the levels of H3K36me2 but not the levels of H3K36me3 and H3K4me1/2/3. Furthermore, *dKdm2* null mutants display a shortened circadian period, while overexpression of dKDM2 lengthens the circadian period. These results show that dKDM2 is involved in regulating circadian clocks but does not play essential roles in normal development, and the major target of dKDM2 *in vivo* is H3K36me2. These results also identify the major function of dKDM2 as an epigenetic regulator of circadian rhythms.

### dKDM2 Is Not an Essential Gene for Viability in *Drosophila*

dKDM2 was initially identified as a subunit of the dRAF complex, which couples H2Aub with demethylation of H3K36me2 during Polycomb group silencing (Lagarou et al., 2008). Subsequently, dKDM2 was shown to demethylate H3K4me3 (Kavi and Birchler, 2009). Given the fundamental role of Polycomb complexes in regulating the body plan in animals and the methylation states of H3K4 and H3K36 in regulating transcription activation and elongation, dKDM2 was expected to be critical for development. However, developmental genetic analyses of multiple *dKdm2* mutant alleles in *Drosophila* suggest that *dKdm2* is not required for normal development (Zheng et al., 2014; Shalaby et al., 2017), and this work, which used two null alleles of *dKdm2*, has further validated this conclusion.

It has been reported that *dKdm2* genetically interacts with *Pc^1^*, *Pc^3^*, *trx^1^*, and *ash1^10^* (Lagarou et al., 2008). Three mutant alleles of *dKdm2* were used in these genetic tests, including *dKdm2^DG12810^*, *dKdm2^EY 01336^*, and *dKdm2^KG04325^*. Of these, *dKdm2^DG12810^* and *dKdm2^KG04325^* were reported to be homozygous lethal alleles, while *dKdm2^EY 01336^* is a homozygous viable allele (Lagarou et al., 2008). In another study, *dKdm2^DG12810^* was shown to genetically interact with the KDM5 homolog *little imaginal disc* (*lid*) (Li et al., 2010). However, these alleles were not validated using molecular and biochemical analyses before their usage. In our previous analyses, the *dKdm2^EY 01336^* and *dKdm2^KG04325^* homozygotes were found to be viable. For both of these alleles, the transposons inserted in the second intron of *dKdm2* gene have no effects on both the mRNA and protein levels of dKDM2 (Zheng et al., 2014). After outcrossing the *dKdm2^EP3093^*, *dKdm2^f02828^*, and *dKdm2^D00170^* mutants, which were initially homozygous lethal, with the wild-type flies for four generations, we have observed that the homozygous individuals carrying these alleles are fully viable, thus these original alleles carry second site lethal mutations (Zheng et al., 2014). Using the same outcrossing strategy, the *dKdm2^DG12810^* homozygous mutants are still lethal at the third instar larval and pupal stages, but the transheterozygous of this allele with other strong *dKdm2* alleles and deletion lines are fully viable (Zheng et al., 2014). These observations suggest that the second site lethal mutation(s) in the *dKdm2^DG12810^* allele are close to the *dKdm2* locus, and thus, cannot be easily removed. Given that the existence of second-site mutations can complicate the interpretation of genetic analyses, it is important to validate mutant alleles, especially when they are being used for the first time.

### Role of dKDM2 in Demethylating Histone H3

The dKDM2 protein has several conserved protein domains, including the JmjC, CXXC-type zinc finger, F-box, and Amn1 (Antagonist of mitotic exit network protein 1) domains (Zheng et al., 2014; Holowatyj et al., 2015). Of these domains, the JmjC domain defines its enzymatic activity as a demethylase. Considerable efforts have been invested into identifying the exact histone markers modified by KDM2 proteins *in vitro* and *in vivo.*

Based on the *in vitro* demethylation assay using modified histone H3 peptides as the substrates followed by mass spectrometry analysis, it has been shown that KDM2A preferentially demethylates H3K36me2, but not H3K36me3, which was validated by forced expression of KDM2A in 293T cells (Tsukada et al., 2006). Similar approaches have been used to show that KDM2A only demethylates H3K36me1 and H3K36me2 but not other methylated peptides such as H3K36me3, H3K4me1/2/3, H3K9me1/2/3, and H3K27me1/2/3 (Williams et al., 2014). Moreover, structural analyses demonstrate that KDM2A specifically demethylates H3K36me2 (Cheng et al., 2014). In contrast, the results from analyses using *in vivo* samples are less clear, largely due to different experimental systems and approaches. Both KDM2A and KDM2B can specifically demethylate H3K36me2 (He et al., 2008; Kottakis et al., 2011; Liang et al., 2012). Overexpression of KDM2A in HeLa cells decreased the levels of H3K36me2, while ectopic expression of KDM2B significantly reduced the H2K4me3 level but has no effects on the levels of H3K4me2, H3K9me3, H3K27me3, H3K36me2, and H3K36me3 (Frescas et al., 2007). However, expression of KDM2B in HEK293 and HeLa cells only reduced the levels of H3K36me2, but not H3K4me3 (He et al., 2008). Expression of KDM2B in mouse embryonic fibroblasts also significantly reduced the levels of H3K36me2 (Liang et al., 2012). Furthermore, the JmjC domain of KDM2B tagged with GST can demethylate H3K4me3 but not H3K36me2 in *in vitro* histone demethylation assay (Janzer et al., 2012). These studies suggest that H3K36me2 is the major target for KDM2A and KDM2B in mammalian cells, and KDM2B can also demethylate H3K4me3.

Depletion of dKDM2 in cultured *Drosophila* S2 cells increases the H3K36me2 level but not the levels of H3K36me1/3 or H3K4me3 (Lagarou et al., 2008). Yet, knocking down *dKdm2* in larvae increased the levels of H3K4me3 but not H3K36me2 (Kavi and Birchler, 2009). In our experiments, we observed mild elevation of the H3K36me1/2/3 levels in trans-heterozygous larvae of deletion lines uncovering the *dKdm2* locus, and we could not detect changes on the global levels of H3K36me1/2/3, H3K4me1/2/3, H3K9me2, and H3K27me2/3 in S2 cells (Zheng et al., 2014). Using a more quantitative method for Western blot, we observed a mild increase in the levels of H3k36me1/2/3 and H3K4me1/2/3 in *dKdm2* null mutant larvae (**Figure 2**). However, when wild-type dKDM2 is overexpressed in larvae, we can detect considerable reduction in the level of H3K36me2 and to a lesser extent, the H3K36me1 level, but not the levels of H3K36me3 and H3K4me1/2/3 (**Figures 3A,B**). These observations are validated by immunostaining these epigenetic markers in wing discs, where the cells in the anterior compartment serve as the internal control (**Figures 3C–E**). Taken together, these different results are likely due to different experimental approaches. Considering all the observations summarized above and the intrinsic strengths and limitations of different methods, it appears that most data support H3K36me2 as the major target of dKDM2. It is also possible that other KDM family members could compensate for the loss of dKDM2. Perhaps, instead of analyzing the global effects of dKDM2 on epigenetic markers, analyzing the levels of these modifications on specific promoters may provide better resolution in the future.

### Role of dKDM2 in Regulating H2A Ubiquitination

The histone H2A lysine 118 (H2AK118) in *Drosophila* is mono-ubiquitinated by Sce (Sex combs extra, or dRING), and its vertebrate orthologs, Ring1 and Ring2/Ring1B, act as the E3 mono-ubiquitin ligase for the corresponding H2A at K119 (de Napoles et al., 2004; Wang et al., 2004; Lagarou et al., 2008; Gutierrez et al., 2012). The dimerization between RING1 and a member of the family of Polycomb group ring finger (PCGF) proteins is necessary for the H2A ubiquitinase activity of RING1 (Wang et al., 2004; Cao et al., 2005). In *Drosophila*, the PCGF proteins include Posterior sex combs (Psc), Suppressor of zeste 2 [Su(z)2], and L(3)73Ah (Dorafshan et al., 2017). The dKDM2 has been identified as a key subunit of the dRAF complex, which is composed of dRING, Psc, dKDM2, RAF2 and Ulp1, and dKDM2 was found to be required for the ubiquitination of H2A by dRING-Psc (Lagarou et al., 2008). However, no obvious change was observed in the overall levels of H2Aub in the *dKdm2* null mutant larvae, which is consistent with a recent report showing that the dRING-L(3)73Ah complex plays a major role in the production of the bulk of ubiquitinated H2A: depletion of the PCGF protein L(3)73Ah, but not Psc and Su(z)2, significantly reduced the H2Aub level in S2 cells (Kahn et al., 2016).

The mammalian KDM2A (also known as FBXL11) and KDM2B (i.e., FBXL10) have a PHD domain; the PHD domain of the mammalian KDM2B can specifically bind to H3K4me3 and H3K36me2 (Janzer et al., 2012). It is interesting to note that the PHD domain is closely related to the RING domains found in E3 ligases (Chasapis and Spyroulias, 2009; Matthews et al., 2009). The PHD domain of the mammalian KDM2B shows the E3 ubiquitin ligase activity, yet KDM2B alone could not ligate H2A with ubiquitin in the *in vitro* ubiquitination assay, suggesting the requirement of other proteins interacting with KDM2B for this activity (Janzer et al., 2012). It is interesting to note that the PHD domain is absent in KDM2B proteins in chickens, frogs, zebra fish, and the invertebrate KDM2 proteins (Zheng et al., 2014). Taken together, these observations suggest that the major function of dKDM2 is to demethylate H3K36me2, and more work is needed to determine its role in targeting other epigenetic marks, particularly on specific promoters.

### dKDM2 Regulates Circadian Rhythms

The circadian clocks enable animals to anticipate daily environmental changes and control most bodily functions including behavior and metabolism. In human osteosarcoma cells, it has been reported that depletion of human KDM2A shortens the circadian period, while its overexpression lengthens the period (Reischl and Kramer, 2015). However, there is little evidence about the roles of KDM2 in circadian regulation at the organismal level. Here, we identified that dKDM2 regulates circadian rhythmicity and period in *Drosophila*. More importantly, our *in vivo* data is consistent with previous studies in human cells: *dKdm2* nulls have a shortened period, and overexpression of dKDM2 lengthened circadian period (**Figure 4** and **Supplementary Table S1**). In the preparation of this manuscript, we noticed that a recent study has also observed that *dKdm2* mutants have shortened period (Shalaby et al., 2018). These results suggest that the roles of KDM2 in circadian rhythms are conserved among species.

How does dKDM2 regulate the circadian period? To test whether dKDM2 controls the molecular pacemaker or circadian locomotor output, we have analyzed the levels of two critical pacemaker proteins, CLK and PER, in constant darkness. However, no obvious changes of these pacemaker proteins were observed, at least at the peak level. Although we cannot exclude potential effects of dKDM2 mutation on other circadian proteins, it is unlikely that dKDM2 affects the molecular pacemaker. Considering the expression of *36Y-GAL4* and the endogenous dKDM2 protein in peptidergic neurons, including in the pars intercerebralis, it is possible that dKDM2 regulates the circadian locomotor output pathway. Taken together, these results suggest that dKDM2 may regulate the circadian clock through an unknown circadian output instead of regulating the levels of the molecular pacemakers such as CLK and PER.

## Conclusion

Our results show that the major role of dKDM2 is the epigenetic regulation of circadian behavior, rather than normal development. Because small chemical inhibitors have been actively developed to specifically target KDMs in recent years (Hoffmann et al., 2012; Thinnes et al., 2014; Morera et al., 2016; Song et al., 2016; Tsai and So, 2017), our results suggest that it will be important to determine whether the KDM2 inhibitors may affect the circadian clocks in both *Drosophila* and mammals. It will be necessary to perform detailed analyses of changes of epigenetic marks in neurons from *dKdm2* mutants, particularly for the genes involved in regulating circadian clocks and other behaviors.

## Author Contributions

J-YJ, YoZ, and J-QN conceived and designed the experiments. YaZ, YX, XR, ML, XL, YJ, and YN performed the experiments. YaZ, YX, XR, J-QN, YoZ, and J-YJ analyzed the data. J-YJ, YoZ and J-QN wrote the paper. All authors approved the publication of this work.

## Conflict of Interest Statement

The authors declare that the research was conducted in the absence of any commercial or financial relationships that could be construed as a potential conflict of interest.

## Footnotes

1 https://dgrc.bio.indiana.edu/product/View?product=1076

2 https://imagej.nih.gov/ij/

## Supplementary Material

FIGURE S1 Characterization of the *UAS-dKdm2^+^-eGFP* line. **(A)** Nuclear localization of dKDM2-eGFP proteins driven by salivary-specific *Sgs3-Gal4* line. **(B)** Analysis of the mRNA levels of *dKdm2* in “*ubi-Gal4/UAS-dKdm2^+^-eGFP*” larvae using qRT-PCR. The regions uncovered by the primers are shown in **Figure 1A**. The Student’s *t*-test was performed between the “*Ubi-Gal4/UAS-dKdm2^+^-eGFP*” line and *w^1118^* (or the “*UAS-dKdm2^+^-eGFP /+*” line), and the ^∗^ above magenta bars represents both sets of the *t*-tests. **(C)** Ectopic expression of eGFP-tagged dKDM2 with the *ubi-Gal4* line (genotypes are the same as color-coded in **B**) assayed by a Western blot.

FIGURE S2 Expression of the endogenous dKDM2 protein tagged with eGFP in adult brain. **(A)** Representative confocal image of dKDM2-eGFP (green), stained together with antibody against PDF (**A′**, blue), and the merged image is shown in **(A″)**. Scale bar in **(A)**: 100 μm. **(B)** Expression of dKDM2 in sLNv, **(B1,B2)** are two successive focal planes. **(C)**Expression of dKDM2 in lLNv, C1, and C2 are two successive focal planes. **B1,B2, C1,C2** for GFP, **B1′,B2′,C1′,C2′** for PDF; **B1″,B2″,C1″,C2″**; show the merged channels. Scale bars in **(B1,B2, C1,C2)**: 10 μm.

FIGURE S3 Locomotor behavior of flies with dKDM2 overexpressed in circadian cells. Representative double plotted actograms showing average activity of flies during 3 days of LD and 5 days of DD. **(A)** Control (*tim-GAL4/+ or* TG4/+). **(B)** Overexpression of dKDM2 in circadian cells; genotype: *TG4/+; UAS-dKdm2-EGFP/+*. White color represents the light phase, and the dark phase is shown in gray.

FIGURE S4 Quantification of PER and CLK levels in *dKdm2* mutants. Bar graph shows the abundance of PER in sLNv CT1 **(A)**, CLK in sLNv CT4 **(B)**, and PDF in sLNv CT1 **(C)**. **(D)** Expression of PER (in red) in lLNv CT1, co-stained with anti-PDF (in green), and the results are quantified and shown in **(D′)**. **(E)** Expression of CLK (in red) in lLNv CT4, co-stained with anti-PDF (in green), and the results are quantified and shown in **(E′)**. ^∗∗^*P* < 0.01 based on Student’s *t*-tests.

TABLE S1 Effects of dKDM2 mutation or overexpression on circadian behaviors.
