# Supplementary figures and images for: The Lysine Demethylase dKDM2 Is Non-essential for Viability, but Regulates Circadian Rhythms in Drosophila
Source: Front Genet. 2018 Sep 4;9:354. doi: 10.3389/fgene.2018.00354 (PMC6131532; doi:10.3389/fgene.2018.00354)

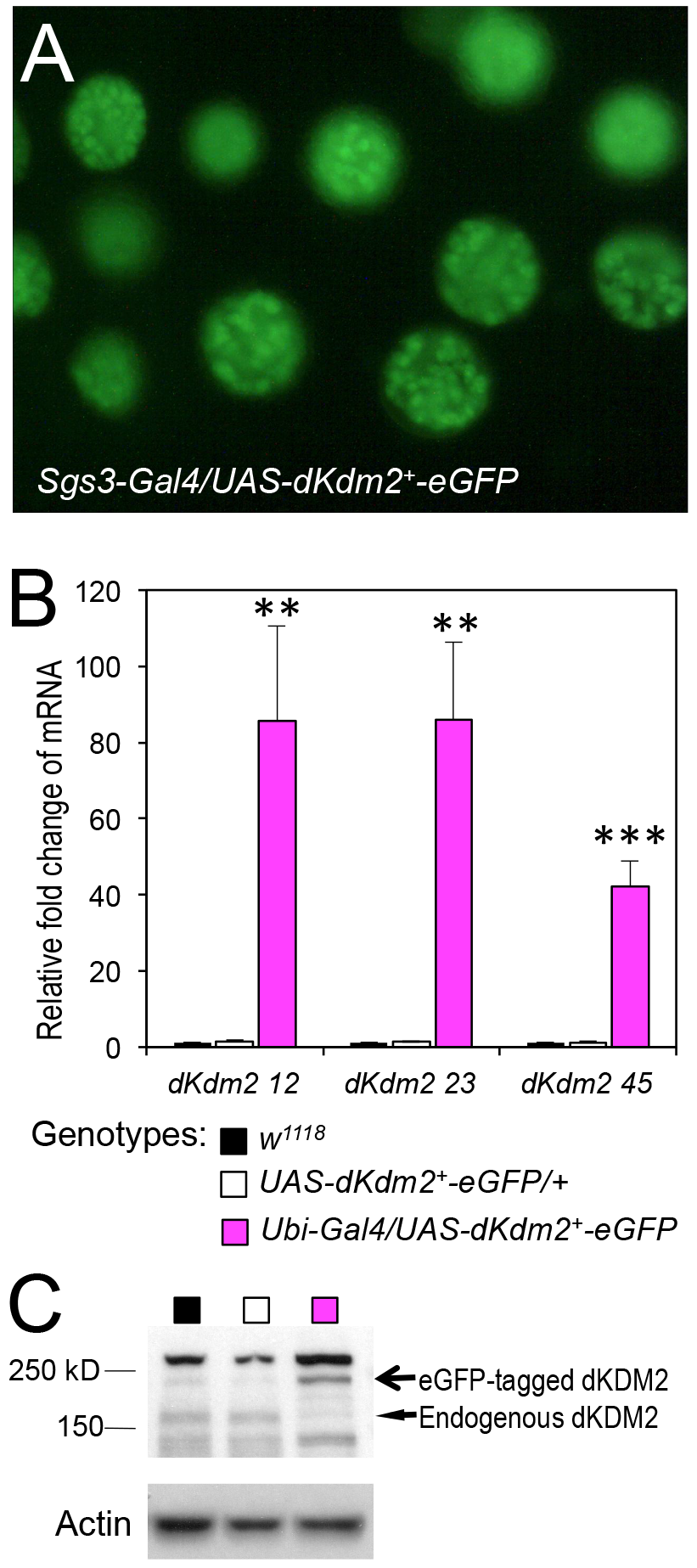

Supplement: FIGURE S1 — Characterization of the UAS-dKdm2+-eGFP line. (A) Nuclear localization of dKDM2-eGFP proteins driven by salivary-specific Sgs3-Gal4 line. (B) Analysis of the mRNA levels of dKdm2 in “ubi-Gal4/UAS-dKdm2+-eGFP” larvae using qRT-PCR. The regions uncovered by the primers are shown in Figure 1A. The Student’s t-test was performed between the “Ubi-Gal4/UAS-dKdm2+-eGFP” line and w1118 (or the “UAS-dKdm2+-eGFP /+” line), and the ∗ above magenta bars represents both sets of the t-tests. (C) Ectopic expression of eGFP-tagged dKDM2 with the ubi-Gal4 line (genotypes are the same as color-coded in B) assayed by a Western blot. [file Image_1.TIF]

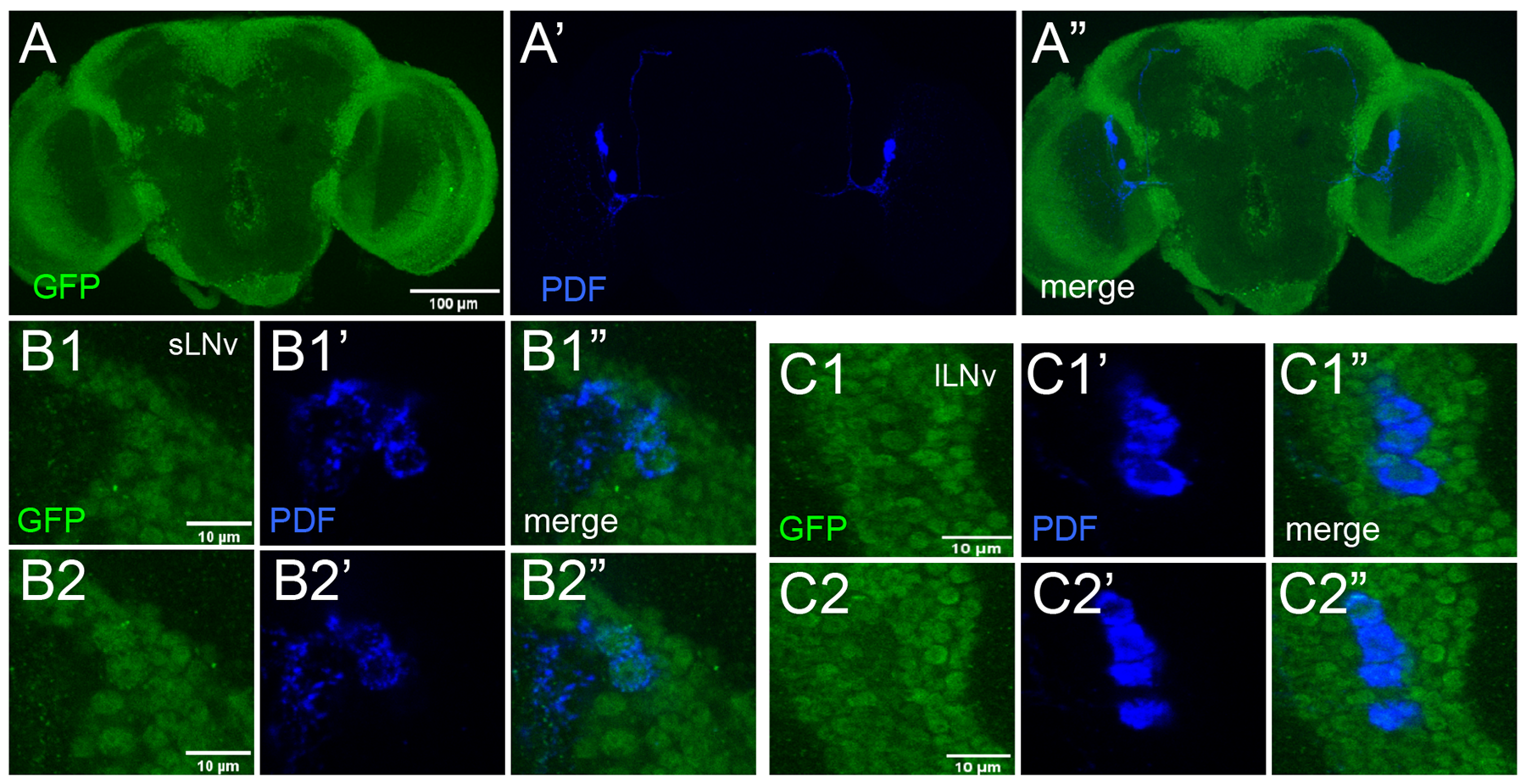

Supplement: FIGURE S2 — Expression of the endogenous dKDM2 protein tagged with eGFP in adult brain. (A) Representative confocal image of dKDM2-eGFP (green), stained together with antibody against PDF (A′, blue), and the merged image is shown in (A″). Scale bar in (A): 100 μm. (B) Expression of dKDM2 in sLNv, (B1,B2) are two successive focal planes. (C)Expression of dKDM2 in lLNv, C1, and C2 are two successive focal planes. B1,B2, C1,C2 for GFP, B1′,B2′,C1′,C2′ for PDF; B1″,B2″,C1″,C2″; show the merged channels. Scale bars in (B1,B2, C1,C2): 10 μm. [file Image_2.TIF]

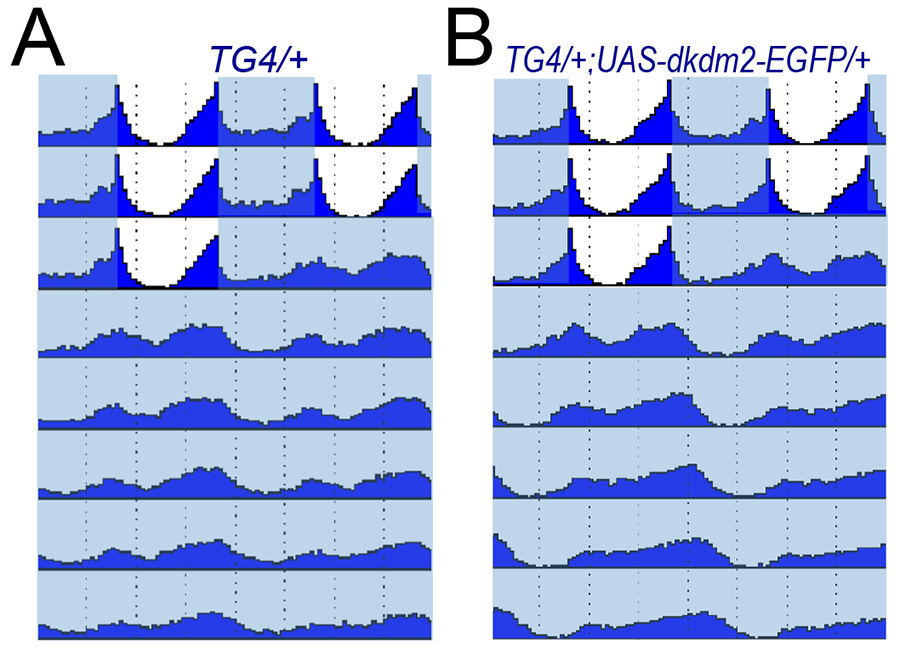

Supplement: FIGURE S3 — Locomotor behavior of flies with dKDM2 overexpressed in circadian cells. Representative double plotted actograms showing average activity of flies during 3 days of LD and 5 days of DD. (A) Control (tim-GAL4/+ or TG4/+). (B) Overexpression of dKDM2 in circadian cells; genotype: TG4/+; UAS-dKdm2-EGFP/+. White color represents the light phase, and the dark phase is shown in gray. [file Image_3.TIF]

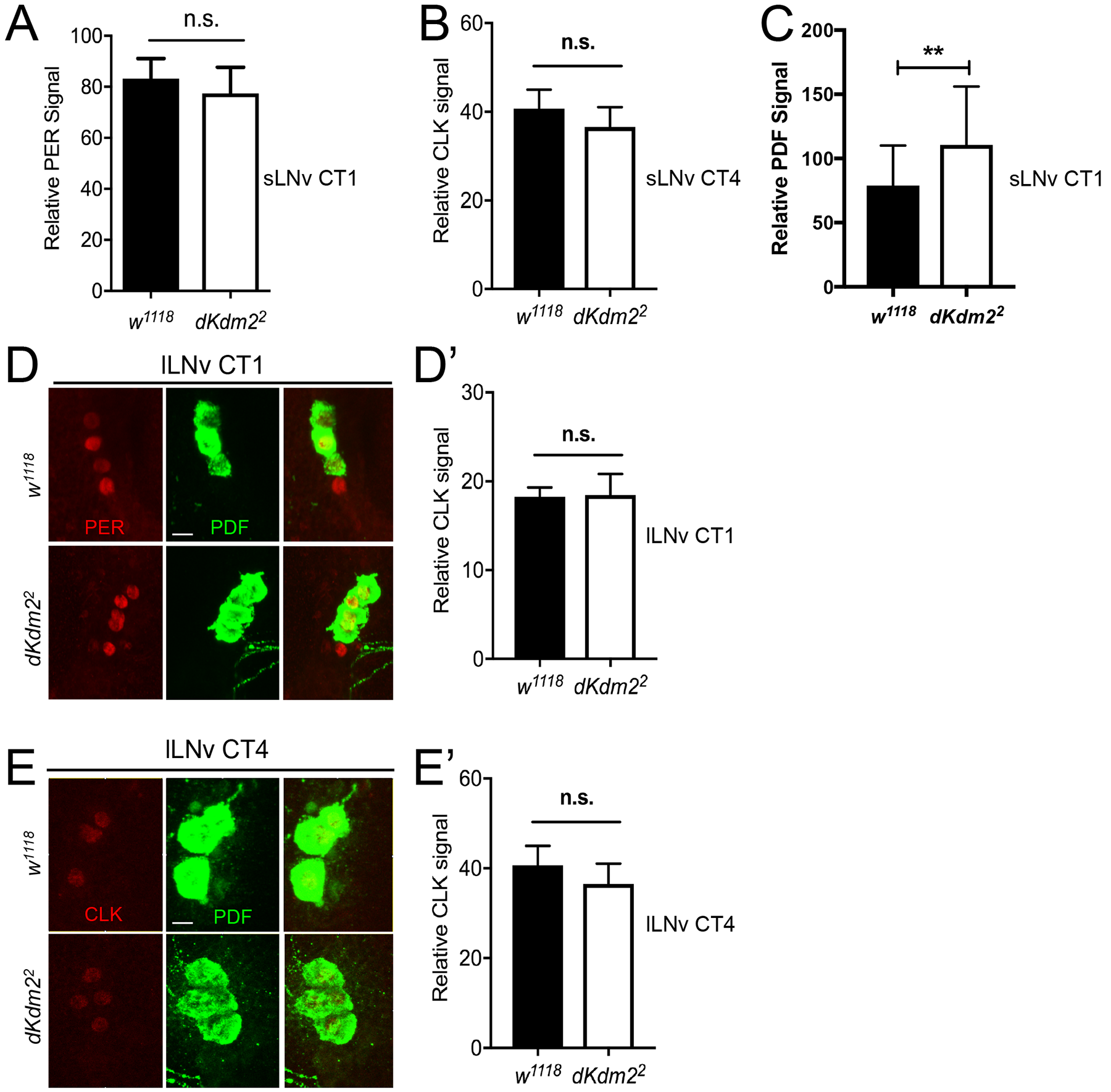

Supplement: FIGURE S4 — Quantification of PER and CLK levels in dKdm2 mutants. Bar graph shows the abundance of PER in sLNv CT1 (A), CLK in sLNv CT4 (B), and PDF in sLNv CT1 (C). (D) Expression of PER (in red) in lLNv CT1, co-stained with anti-PDF (in green), and the results are quantified and shown in (D′). (E) Expression of CLK (in red) in lLNv CT4, co-stained with anti-PDF (in green), and the results are quantified and shown in (E′). ∗∗P < 0.01 based on Student’s t-tests. [file Image_4.TIF]
