# Supplementary material for: The Lysine Demethylase dKDM2 Is Non-essential for Viability, but Regulates Circadian Rhythms in Drosophila
Source: Front Genet. 2018 Sep 4;9:354. doi: 10.3389/fgene.2018.00354 (PMC6131532; doi:10.3389/fgene.2018.00354)
Supplement: TABLE S1 — Effects of dKDM2 mutation or overexpression on circadian behaviors. [file Table_1.pdf]

Table S1. Effects of dKDM2 mutation or overexpression on circadian behaviors

Genotypes	Period (h)	Rhythmicity	Power	N
w^{1118}	24.9±0.11	87.00%	64.6±4.01	54
$dKdm2^{2}$	23.5±0.14	59.30%	44.3±3.82	59
$dKdm2^{1}$	23.8±0.16	37.00%	44.0±5.04	54
$dKdm2^{1}$/ $dKdm2^{2}$	23.8±0.11	81.00%	69.7±6.18	42
TG4/+	24.6±0.06	90.10%	74.4±3.55	71
UAS-dKdm2-EGFP/+	23.8±0.16	100%	66.7±4.20	16
TG4/UAS-dKdm2-EGFP	25.7±0.06	94.70%	82.4±2.16	75
PG4/+	24.7±0.08	76.20%	69.3±3.82	42
PG4/UAS-dKdm2-EGFP	25.6±0.06	96.70%	78.6±2.75	61
TG4/+;pdf-Gal80/+	24.1±0.09	91.20%	70.1±5.57	34
TG4/+; pdf-Gal80/UAS-dKdm2-EGFP	24.7±0.08	92.30%	79.1±4.20	26
